# The Inflammatory Feed-Forward Loop Triggered by the Complement Component C3 as a Potential Target in Endometriosis

**DOI:** 10.3389/fimmu.2021.693118

**Published:** 2021-08-13

**Authors:** Chiara Agostinis, Sonia Zorzet, Andrea Balduit, Gabriella Zito, Alessandro Mangogna, Paolo Macor, Federico Romano, Miriam Toffoli, Beatrice Belmonte, Gaia Morello, Anna Martorana, Violetta Borelli, Giuseppe Ricci, Uday Kishore, Roberta Bulla

**Affiliations:** ^1^Institute for Maternal and Child Health, IRCCS Burlo Garofolo, Trieste, Italy; ^2^Department of Life Sciences, University of Trieste, Trieste, Italy; ^3^Tumor Immunology Unit, Human Pathology Section, Department of Health Sciences, University of Palermo, Palermo, Italy; ^4^Department of Health Promotion, Mother and Child Care, Internal Medicine and Medical Specialties, University of Palermo, Palermo, Italy; ^5^Department of Medical, Surgical and Health Science, University of Trieste, Trieste, Italy; ^6^Biosciences, College of Health, Medicine and Life Sciences, Brunel University London, Uxbridge, United Kingdom

**Keywords:** C3, endometriosis, mast cells, complement system, TNF-α

## Abstract

The complement system is a major component of humoral innate immunity, acting as a first line of defense against microbes *via* opsonization and lysis of pathogens. However, novel roles of the complement system in inflammatory and immunological processes, including in cancer, are emerging. Endometriosis (EM), a benign disease characterized by ectopic endometrial implants, shows certain unique features of cancer, such as the capacity to invade surrounding tissues, and in severe cases, metastatic properties. A defective immune surveillance against autologous tissue deposited in the peritoneal cavity allows immune escape for endometriotic lesions. There is evidence that the glandular epithelial cells found in endometriotic implants produce and secrete the complement component C3. Here, we show, using immunofluorescence and RT-qPCR, the presence of locally synthesized C3 in the ectopic endometriotic tissue, but not in the eutopic tissue. We generated a murine model of EM *via* injection of minced uterine tissue from a donor mouse into the peritoneum of recipient mice. The wild type mice showed greater amount of cyst formation in the peritoneum compared to C3 knock-out mice. Peritoneal washings from the wild type mice with EM showed more degranulated mast cells compared to C3 knock-out mice, consistent with higher C3a levels in the peritoneal fluid of EM patients. We provide evidence that C3a participates in an auto-amplifying loop leading to mast cell infiltration and activation, which is pathogenic in EM. Thus, C3 can be considered a marker of EM and its local synthesis can promote the engraftment of the endometriotic cysts.

**Graphical Abstract:**
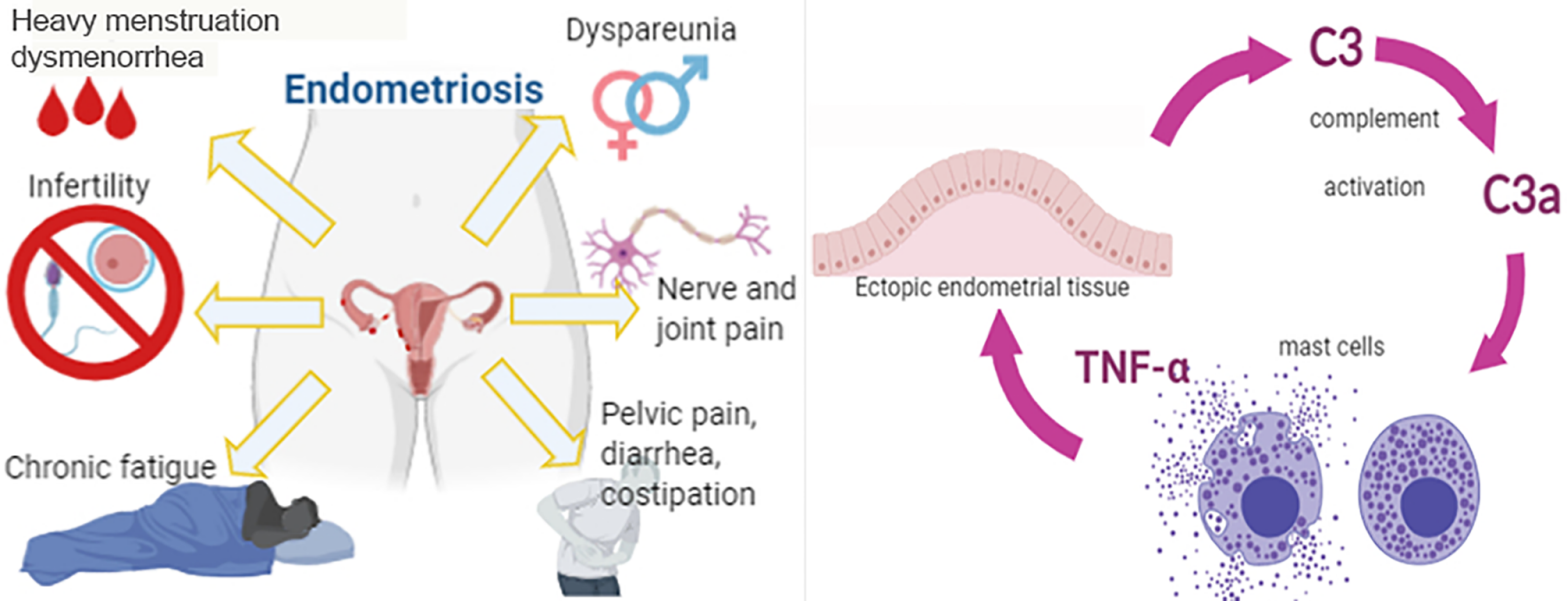


## Introduction

Endometriosis (EM) is a chronic gynecological disorder, frequently associated with infertility, that affects about 5-10% of women in reproductive age (1, 2). EM is characterized by severe pain, dysmenorrhea, dyspareunia and dysuria, as a consequence of the presence of functional endometrial tissue outside the uterine cavity (3). The most common locations for the ectopic implants are the ovaries, peritoneum, and the utero-sacral ligaments. The presence of ectopic tissues in these areas induces a condition of chronic inflammation. Current evidence suggests that immune dysfunction is the most likely causative factor for the EM pathogenesis (4–6). In particular, the pathways involved in immune cell recruitment, cell adhesion, and inflammatory processes encourage the implantation and survival of endometriotic lesions.

The current consensus is that EM involves a local pelvic inflammatory process with altered functions of immune-related cells in the peritoneal environment (7). Recent studies suggest that the peritoneal fluid (PF) of women with EM contains an increased number of activated macrophages that secrete various humoral mediators locally, including growth factors, cytokines and possibly, oxidative products (8), leading to the development and progression of EM and EM-associated infertility (9). Although the contributions of specific immune cell subsets and their mediators to the onset and the course of the inflammatory process in endometrial lesions are still poorly understood, evidence suggests that mast cells (MCs) are crucially involved in the inflammatory process associated with EM. In fact, high numbers of degranulated MCs have been found in endometriotic lesions (10–12).

Recently, it has been shown that one of the most predominant pathways altered in EM is the complement system (13, 14). The complement system is an important part of the innate immunity and acts as a bridge between innate and adaptive immune system. It is involved in host defense against infectious agents and altered self. Three different molecules are responsible for the recognition phase of complement: C1q, Mannose-Binding Lectin (MBL), and C3. The binding of the recognition molecules to the target ligands initiates the three different complement pathways: the classical (*via* C1q), alternative (*via* C3) and lectin (*via* MBL) pathway (15, 16). The three pathways eventually converge in the formation of the C3 and C5 convertases. This then results in the generation of the main effector molecules of the complement system: the opsonins C3b and C4b, the anaphylatoxins C3a, C4a and C5a, and the Membrane Attack Complex (MAC) that causes the target cell lysis. The small complement fragments, anaphylatoxins, cause local inflammatory responses by acting, for instance, on MCs, inducing an increase in blood flow, vascular permeability, and leukocyte recruitment (17).

The presence of the complement component C3 in endometriotic tissues has been reported since 1980 (18, 19). However, from 1980 to date, no significant progress has been made in understanding the reasons for C3 presence in the endometriotic microenvironment and the role it played in the development of lesions. All currently available therapies for EM are not etiological but symptomatic treatments, accounting just for a partial and transitory relief of the symptoms (20). The prevalent therapeutic options for EM-associated pain are represented by contraceptive rather than fertility-promoting treatments (21). Immunotherapy is beginning to be considered as an option for EM. In the light of the possibility to use complement immunotherapy with blocking antibodies in EM (22), we examined the underlying mechanisms of C3 expression and its cellular cross-talk, making it a novel potential therapeutic target for EM.

The pathophysiology of EM is an excellent example of immune dysfunction reminiscent of tumour microenvironment, and strongly connected with infertility. Here, we aimed to investigate the interplay between C3 and MCs, and their involvement in the pathogenesis of EM, through the development of *in vitro* and *in vivo* models.

## Materials and Methods

### Patients

Patients and control women were enrolled at the Institute for Maternal and Child Health, IRCCS Burlo Garofolo, Trieste, Italy. The study was reviewed and approved by the Regional Ethical Committee of FVG (CEUR), Udine, Italy (Prot. 0010144/P/GEN/ARCS 2019). Informed consent for participation in the study was obtained from all participants. The study group consisted of a total of 7 women, diagnosed with moderate/severe and minimal/mild EM, according to the revised criteria of the American Society for Reproductive Medicine (23).

### Cell Lines

HepG2, AN3CA and THP-1 cell lines were obtained from American Type Culture Collection (ATCC). HMC-1 cells were kindly provided by Prof. Carlo Pucillo (University of Udine, Italy). AN3CA and HepG2 cells were cultured in DMEM/F12, HMC-1 and THP-1 in RPMI-1640, both media were supplemented with 10% v/v FBS. THP-1 cells were differentiated into macrophage-like phenotype by adding 15 ng/mL of Phorbol 12-Myristate 13-Acetate (PMA; Sigma) in RPMI-1640 complete medium for 48 h at 37°C in 5% v/v CO_2_. The medium always contained 100 U/mL penicillin and 100 μg/mL of streptomycin.

### Primary Cell Isolation and Culture

Primary endometriotic cells were isolated from EM patient lesions. Briefly, tissues were digested overnight (ON) at 4°C with 0.25% trypsin (Sigma-Aldrich), 50 μg/mL DNase 1 (Roche, Milan, Italy) in PBS and then treated with collagenase type I (3 mg/mL; Worthington Biochemical) for 30 min at 37°C. As described earlier (24), endothelial cells (EECs) were positively selected with Dynabeads M-450 (Life Technologies, Milan, Italy) coated with Ulex europaeus 1 lectin (Sigma-Aldrich), seeded on 12,5 cm^2^ flask precoated with 2 µg/cm^2^ fibronectin (Roche). Cells were maintained in serum-free endothelial basal medium (Life Technologies, Monza, Italy), supplemented with 20 ng/mL bFGF (basic Fibroblast Growth Factor), 10 ng/mL EGF (Epidermal Growth Factor), 10% v/v FBS (all from Life Technologies), and 10% v/v heat inactivated human serum and incubated at 37°C, 5% CO_2_. Endometriotic epithelial/stromal cells (EM cells), obtained *via* the negative selection, were cultured in the same medium containing only 10% FBS.

Moreover, uterine microvascular endothelial cells (UtMECs) were isolated from normal uterus of healthy women, following the same procedure.

### Murine Model of EM

C57BL/6 WT mice were purchased from Harlan Laboratories. *C3*^-/-^ mice were kindly provided by Prof. Marina Botto, Centre for Complement and Inflammation Research, Department of Medicine, Imperial College, London, UK and generated as described previously (25). All animals were handled in accordance with the institutional guidelines and in compliance with the European (86/609/EEC) and the Italian (D.L.116/92) laws. The Institutional Animal Care Committee of the University of Trieste approved the procedures (Prot. 35/2010B). The mouse model of EM was adapted from Somigliana and Mariani (26, 27). Briefly, donor mice were injected with 17-β-estradiol-3-benzoate (Sigma-Aldrich; 100 µg/Kg i.m.) and sacrificed 1 week later; the uterus was removed, the two horns were isolated, the myometrium was removed by scraping, and the remaining endometrial tissue was reduced to small fragments with scissors. The fragments derived from the isolated uterine horns were weighed and suspended in 400 µl saline (1 mg/ml); half of the preparation was injected into the peritoneum of each of two recipient mice with a syringe (Day 0). Carprofen (5 mg/kg body weight) was given as an analgesic immediately after the surgery and again after 48 h. Hormonal therapy with 17-β-estradiol-3-benzoate (Sigma-Aldrich, 50 µg/kg i.m.) was initiated at the time of tissue injection and at 2-day interval thereafter. Mice were sacrificed on day 21 *via* administration of a lethal dose of anesthetic, their abdomen was opened, and the presence of the lesions was evaluated by an operator blinded to the different conditions. Translucid isolated or grouped superficial lesions were mainly found on the abdominal wall, on the epiploon and around the uterus. Deeply infiltrating lesions were never observed in this model. In some cases, lesions resembling human chocolate cysts were found.

### Characterization of EM Cells

EM cells were plated on 8-chamber culture slides (BD Biosciences Discovery Labware, Milan, Italy). Cells, when grown to confluence, were fixed and permeabilized with FIX & PERM (Società Italiana Chimici, Rome, Italy). Next, cells were incubated with primary monoclonal antibody (mAb) (clone 9) mouse anti-human vimentin (Sigma-Aldrich), (cloneF8/86) mouse anti-human vWF (Dako-Cytomation, Milan, Italy), or mouse anti-human CK 8/18 (Abcam, Italia, Milan, Italy) for 1 h at room temperature (RT), followed by fluorescein isothiocyanate (FITC)-conjugated goat anti-mouse IgG for 1 h at RT. Images were acquired using Leica DM3000 microscope (Leica, Wetzlar, Germany) and collated using a Leica DFC320 digital camera (Leica).

### Gene Expression Analysis

RNA was extracted from cells using kit supplied by Norgen Biotek Corp. (Aurogene, Rome, Italy) according to the supplier’s instructions and reverse transcripted to cDNA through SuperMix kit (Bioline). qPCR was carried out on a Rotor-Gene 6000 (Corbett, Qiagen, Milan, Italy) using SYBR™ Green PCR Master Mix (Applied Biosystems, Milan, Italy). **Supplementary Table 1** shows the primers used for RT-qPCR. The melting curve was recorded between 55°C and 99°C with a hold every 2s. The relative amount of gene expression in each sample was determined by the Comparative Quantification (CQ) method supplied as a part of the Rotor Gene 1.7 software (Corbett Research) (28). The relative amount of each gene was normalized with 18S and expressed as arbitrary units (AU), considering 1 AU obtained from HepG2 cells as a calibrator.

### Western Blot Analysis

10^6^ AN3CA cells seeded onto 6-well plates were treated with 100 ng/mL of TNF-α or 5 ng/mL of IL-1β for 36 h. Cell lysates were fractioned by 10% SDS-PAGE under reducing conditions and transferred to a nitrocellulose membrane using the semi-dry transfer apparatus Trans-Blot Turbo System according to the manufacturer’s protocol (BIO-RAD). 100 ng of recombinant human C3 (Quidel) was used as a control. After 1 h of incubation with 5% skimmed milk in TBST (10 mM Tris, pH 8.0, 150 mM NaCl, 0.5% Tween 20), the membrane was probed with 1:500 anti-C3 (MyBioSource) ON at 4°C. Membrane was washed three times for 5 min and incubated with 1:10000 anti-goat LI-COR IRDye 800CW for 1h at RT. After three washing steps, the fluorescence intensity was acquired by the Odyssey^®^ CLx near-infrared scanner (LI-COR Biosciences, Lincoln, NE, USA). Image acquisition, processing and data analysis were performed with Image Studio Ver 5.2 (LI-COR Biosciences).

### Measurement of C3a

The levels of C3a in PFs from EM patients (n = 16) and from control patients (n = 8), and C3 in cell culture supernatant were evaluated by ELISA kits purchased from Quidel.

### Co-Culture and Cell Stimulation

A confluent 24 well-plate of AN3CA cells was stimulated ON with 100 ng/mL of TNF-α, or 5 ng/mL of IL-1β (both from PeproTech, ListerFish, Milan, Italy), or 10% of pooled EM-PF. For the co-culture study, the cells were seeded at confluence, in the lower part of a 1µm pore TW system (Corning, Milan, Italy), whereas HMC-1 or THP-1 (2x10^5^/TW) were present in the upper part, in the presence of 10% of pooled EM-PF, with or without blocking anti-human C3a (clone H13, 20 µg/mL; MyBiosource, Aurogene, Milan, Italy) (29). Subsequently, the cells were lysed for RNA extraction, the supernatant recovered and stored at -80°C.

### Immunohistochemical Analysis

Uterine and ectopic endometrial tissues were used in this study, after approval by the University Hospital of Palermo Ethical Review Board (approval number 09/2018). Our study selected two cases of proliferative and secretory endometrium as controls, and two cases of patients with tubal and abdominal wall endometriosis.

Immunohistochemistry (IHC) was performed using a polymer detection method. Briefly, tissue samples were fixed in 10% v/v buffered formalin and then paraffin embedded. 4 µm-thick tissue sections were deparaffinized and rehydrated. The antigen unmasking technique was performed using Novocastra Epitope Retrieval Solutions, pH 6 EDTA-based (Leica Biosystems) in thermostatic bath at 98°C for 30 min. Sections were then brought to RT and washed in PBS. After neutralization of the endogenous peroxidase with 3% v/v H_2_O_2_ and Fc blocking by a specific protein block (Novocastra, Leica Biosystems), samples were incubated for 1 h at RT with mouse anti-human C3a/C3a (dilution 1:50, pH 6) monoclonal antibody (Millipore), mouse anti-human C3aR (D-12; dilution 1:50, pH 6) monoclonal antibody (Santa Cruz) and rabbit anti-human C3 (dilution 1:200, pH 9) polyclonal antibody (Sigma-Merk). Staining was revealed *via* polymer detection kit (Novocastra, Leica Biosystems) and AEC (3-amino-9-ethylcarbazole), or DAB (3, 3’-diaminobenzidine) substrate chromogen, both purchased from Dako (Denmark). Slides were counterstained with Harris Hematoxylin (Novocastra, Leica Biosystems). Toluidine blue stain was carried out to detect the presence of mast cells in uterine and ectopic endometrial tissues, according to the manufacturer’s kit Histoline. Slides were analyzed under the Axio Scope A1 optical microscope (Zeiss) and microphotographs were collected through the Axiocam 503 color digital camera (Zeiss) using the Zen2 software. Quantitative analyses of C3 IHC staining were performed by calculating the average percentage of positive signals in five non-overlapping fields for each sample (normal endometrium, n = 3; ovarian EM, n = 3; adnexal EM, n = 3; peritoneal EM, n = 3) at medium-power magnification (200x), using the Positive Pixel count v9, ImageScope software.

### Tryptase Concentration and Enzymatic Activity

Tryptase ELISA kit (USCN, Life Sciences Inc) was used to determine the concentration of tryptase in mice PF samples. The assays were performed according to the manufacturer’s instructions and the results referred to a calibration curve expressed in ng/mL. Samples were assayed in triplicate.

### Gene Expression Profiling (GEP) Analysis

The expression levels of C3 and C3AR1 genes in control endometrium and various EM lesions (peritoneal, deep and ovarian EM) were analyzed using data extracted from the GEO (Gene Expression Omnibus of the National Center for Biotechnology Information-NCBI) with the series accession number GSE141549. Microarray analysis on samples obtained from 43 endometrium biopsies of healthy woman, 101 endometrium biopsies of EM patients and 190 EM lesions, and data normalization, have been described by Gabriel and colleagues (30).

### Statistical Analysis

Data were analyzed by GraphPad Prism software 5.0 (GraphPad Software Inc., La Jolla, CA, USA). Unpaired two-tailed Mann-Whitney test was used for the analysis of different tissue C3 gene expression and cells and for GEP analysis; Wilcoxon test was applied for AN3CA stimulation and mouse model. Results were expressed as mean ± SEM of three independent experiments performed in duplicate. P-values <0.05 were considered statistically significant.

## Results

### C3 Is More Abundant in Ectopic, Compared to Eutopic, Endometrium and Is Locally Expressed by Endometriotic Cells

**Figure 1:**
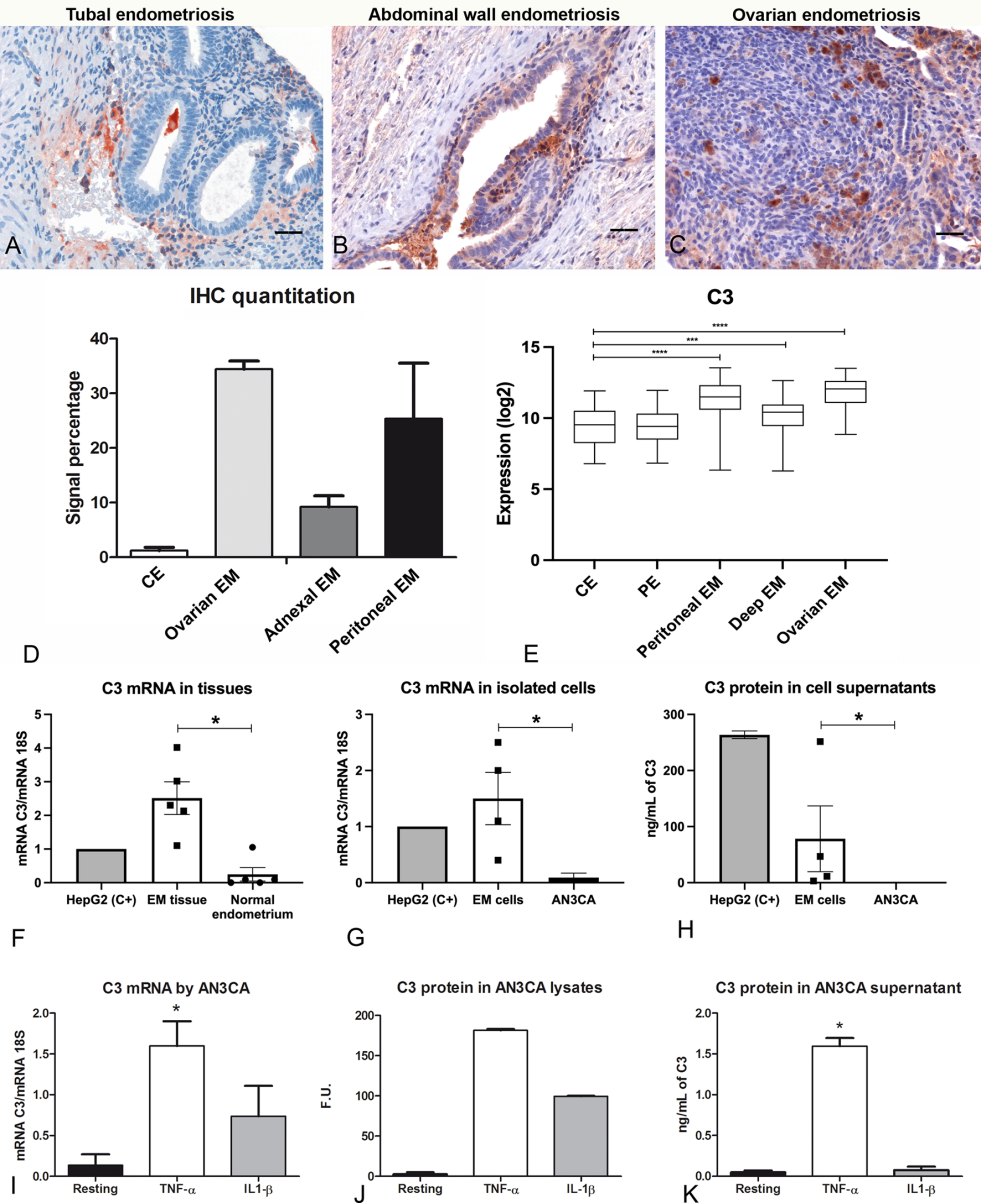
C3 is locally produced in human endometriotic tissue, which is up-regulated by pro-inflammatory cytokines. Representative microphotographs showing expression of C3 by IHC in tubal **(A)**, abdominal wall **(B)** and ovarian **(C)** endometriosis (EM). AEC (red) chromogen was used to visualize the binding of anti-human C3 antibodies. Nuclei were counterstained in blue with Harris Hematoxylin; scale bars, 50μm. **(D)** Quantitative analyses of C3 IHC staining were carried out by calculating the average percentage of positive signals in five non-overlapping fields for each sample (normal endometrium, n=3; ovarian EM, n=3; adnexal EM, n=3; peritoneal EM, n=3) at medium-power magnification (200 x), using the Positive Pixel count v9, ImageScope software, as compared to control endometrium (CE). Data are expressed as mean ± standard deviation. **(E)** Histogram representing C3 mRNA expression in CE, patient endometrium (PE) and in different EM lesions (peritoneal, deep and ovarian EM). GEP analysis, based on data extracted from GEO (GSE141549), highlighted a significantly higher expression of C3 in EM lesions as compared to CE. ***p < 0.001, ****p < 0.0001 (Mann-Whitney U Test). **(F)** The gene expression of C3 by EM tissue (n = 5) was investigated by RT-qPCR and compared to normal endometrium (n= 5) using HepG2 hepatocyte cells as calibrator (AU = 1). 18S was used as the housekeeping gene. Data are expressed as mean of at least three independent experiments ± standard error. *p < 0.05 (Mann-Whitney U Test). **(G)** The C3 mRNA expression was measured by RT-qPCR in EM cells (n= 4) and compared to a normal endometrial cell line, AN3CA. Data are expressed as mean of at least three independent experiments ± standard error. *p < 0.05 (Mann-Whitney U Test). **(H)** Protein levels of C3 were assessed by ELISA in the supernatant of EM cells (n=4) or AN3CA maintained in culture for 60 h. Data are expressed as mean at least three independent experiments± standard error. **(I)** The stimulation of AN3CA cells with pro-inflammatory cytokines induced an up-regulation of C3 expression. AN3CA cells were ON stimulated with TNF-α (100 ng/mL) or IL-1β (5 ng/mL) and the C3 expression was analyzed by RT-qPCR. Data are expressed as mean of at least three independent experiments conducted in duplicate ± standard error. *p < 0.05 (Wilcoxon matched pairs test). **(J)** The expression of C3 was examined in cell lysates by western blot analysis and the intensity of the bands was measured with Odyssey-LICOR scanner. **(K)** Measurement of C3 protein level by ELISA in AN3CA cell culture supernatant stimulated for 36h with TNF-α (100ng/mL) or IL-1β (5ng/mL). Data are expressed as mean of three independent experiments conducted in duplicate ± standard error. *p < 0.05.

We initially confirmed the presence of C3 in human endometriotic tissue sections by immunofluorescence (IF, **Supplementary Figure 1**) and by immunohistochemical assays (IHC, **Figure 1**). IHC, using an anti-human C3 polyclonal antibody, showed moderate cytoplasmic expression of C3 by endometrial stromal cells, and rarely in some glandular epithelial cells in proliferative as well as secretory normal endometrium (**Supplementary Figure 2**). C3 was found to be widely distributed in the EM tissue, with variable intensity, ranging from low in tubal EM, intermediate in abdominal wall EM and high in ovarian EM (**Figures 1A–D**). C3 positivity was mostly localized in the glandular-like structures (**Supplementary Figure 3**; red arrows) and in the cytogen stroma (**Supplementary Figure 3**; green arrows). As reported in **Figure 1E**, the lower C3 expression in healthy or patient derived endometrium as compared to EM lesions was confirmed *via* GEP analysis.

In order to confirm the local synthesis of C3, total RNA was isolated from ovarian EM cysts and eutopic endometrium. C3 gene expression was then analyzed by RT-qPCR, highlighting higher expression levels of C3 transcript in ectopic EM tissues as compared to normal endometrium (**Figure 1F**). HepG2 (hepatocyte cell line) was used as a calibration control.

To assess the contribution of EM epithelial cells to the local C3 production, we isolated EM cells from human ovarian EM cysts, showing marked positivity for both epithelial (cytokeratin 8/18) and stromal (vimentin) markers (**Supplementary Figure 4**). RT-qPCR was performed to evaluate C3 expression differences in isolated EM epithelial cells and AN3CA cells, as a control of normal epithelial endometrial cells. Interestingly, EM cells expressed a higher amount of C3 compared to AN3CA (**Figure 1G**). In addition, only EM cells secreted the C3 protein in the culture supernatant, as measured *via* ELISA (**Figure 1H**).

Since the IHC analysis also revealed a marked positivity in the EM vessels, the expression of C3 was also investigated in EECs and corresponding eutopic cells (UtMECs). We observed a very low expression of C3 transcript by both endothelial cell types (**Supplementary Figure 5**).

### Pro-Inflammatory Stimuli Induce C3 Expression by Normal Endometrial Cell Lines in an *In Vitro* Model of EM

In order to mimic the pro-inflammatory EM microenvironment, we performed *in vitro* experiments, stimulating the endometrial cell line AN3CA with pro-inflammatory cytokines. Our results demonstrated that normal endometrial cells were able to produce a very limited amount of C3 in resting conditions; however, when cells were stimulated with TNF-α, or to a lesser extent with IL-1β, we detected an increase in C3 mRNA levels by RT-qPCR (**Figure 1I**) and C3 protein levels by western blot (**Figure 1J** and **Supplementary Figure 6**). We also found that TNF-α stimulation was able to increase the level of secreted C3 protein, as measured by ELISA in cell supernatant (**Figure 1K**). Furthermore, we demonstrated that the stimulation of C3 transcription induced by TNF-α was dose-dependent (**Supplementary Figure 7**).

### *C3*^-/-^ Mice Are Refractory to Developing EM Cysts in a Syngeneic *In Vivo* Model of EM

**Figure 2:**
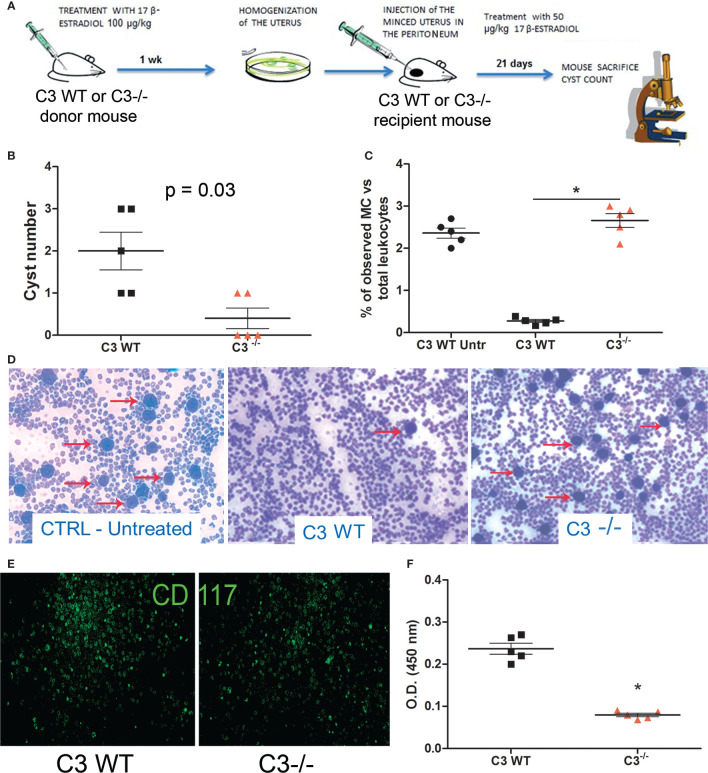
*In vivo* syngeneic mouse model of EM. **(A)** Treatment regimen of C3 WT and gene-deficient mice for generating EM in *vivo* model. Five *C3*^-/-^ and WT mice each were injected (i.p.) with minced uterus of a donor mouse *C3*^-/-^ and WT respectively; after 3 weeks, the peritoneal cyst formation was evaluated. **(B)** Number of cysts counted in wild-type (WT) mice injected with WT endometrium (*n* = 5) or *C3*^-/-^ injected with *C3*^-/-^ endometrium (*n* = 5). Mann-Whitney test p = 0.03. **(C, D)** Representative images of cytocentrifuged peritoneal washing of untreated WT, EM-induced WT and *C3*^-/-^ mice (respectively), stained with Giemsa and counted with ImageJ software (Particle Analysis Tool) to obtain relative percentage between total leukocytes and mast cell (MC)/basophil number. MCs/basophils are identified as blue big dots indicated by red arrows. Original magnification 100×. **(E)** Representative images of cytocentrifuged peritoneal lavage of EM-induced WT mice stained with FITC-conjugated anti-mouse CD117. Original magnification 100×. **(F)** Biochemical characterization of tryptase enzyme present in peritoneal lavage of WT *vs C3*^-/-^ mice by ELISA. *p < 0.05.

To further investigate the role of C3 in EM pathogenesis, we set up a syngeneic model of EM in C57BL/6 WT or C3 gene-deficient (*C3*^-/-^) mice. Estrus was induced in donor animals *via* administration of estradiol. Then, the minced uterus of the donor mice was injected in the peritoneum of recipient mice (**Figure 2A**). After 3 weeks, the animals were sacrificed, and the peritoneal cysts were counted. WT mice developed a higher number of cysts compared to C3 deficient mice (**Figure 2B**).

### Peritoneal Washings Isolated From WT Mice With EM Present More Degranulated MCs Compared to *C3*^-/-^ Mice

We then analyzed the peritoneal washings of WT and *C3*^-/-^ mice for the presence of infiltrating leukocytes. The samples were cytocentrifuged and the cells were stained with Giemsa. The number of infiltrating leukocytes observed in the peritoneal washings of both WT and *C3*^-/-^ treated mice was comparable with those observed in WT untreated (CTRL Untr) mice. A similar number of total peritoneal MCs at baseline between untreated WT and C3^-/-^ mice has been already demonstrated by Prodeus and colleagues (31). Surprisingly, the fluids collected from the WT treated animals contained a lower number of MCs, compared to the *C3*^-/-^ treated mice (**Figures 2C, D**). Next, we stained the cytocentrifuged leucocytes with a monoclonal antibody to CD117, a marker of mature MCs. The peritoneal washings collected from the WT mice contained CD117 positive cells as well (**Figure 2E**). To confirm whether the MCs present in WT peritoneal washings were degranulated, we measured the levels of the MC enzyme, tryptase, in the murine peritoneal washings. The ELISA demonstrated that WT mice with EM had a higher amount of tryptase compared to *C3*^-/-^ or CTRL mice (**Figure 2F**).

### C3a Level Is Higher in the PF of EM Patients, Which Can Act on EM MCs

**Figure 3:**
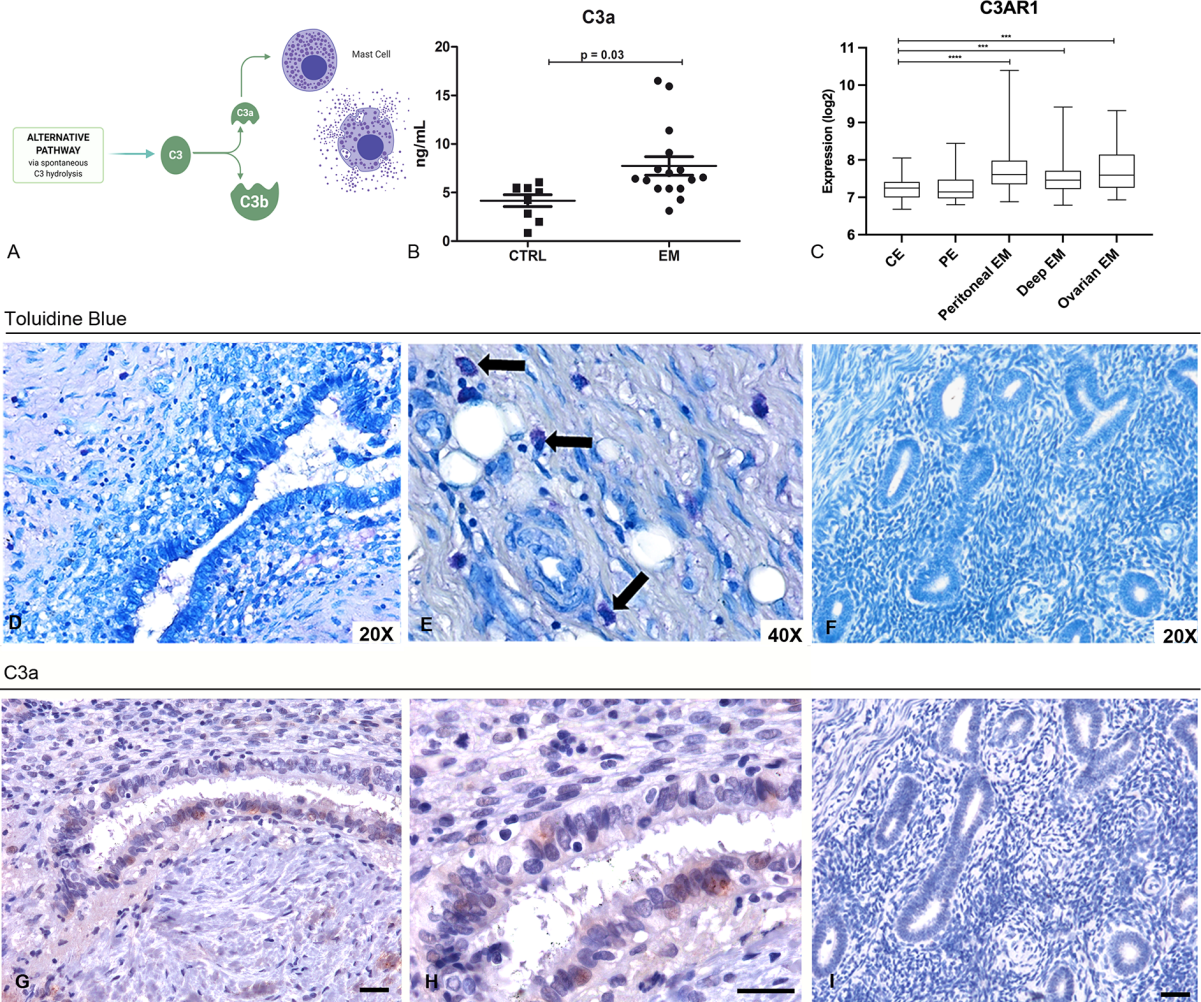
Peritoneal Fluids (PFs) derived from EM patients contained elevated levels of C3a that likely acts on MCs in the EM tissue. **(A)** Graphical representation of the C3 cleavage and C3a formation. **(B)** C3a ELISA evaluation of PFs isolated from EM patients (n = 16) compared to control patient group (n = 8). **(C)** Histogram representing C3AR1 mRNA expression in control endometrium (CE), patient endometrium (PE) and in different EM lesions (peritoneal, deep and ovarian EM). GEP analysis, based on data extracted from GEO (GSE141549), highlighted a significantly higher expression of C3AR1 in EM lesions as compared to CE. ***p < 0.001, ****p < 0.0001 (Mann-Whitney U Test). **(D, E)** Toluidine blue staining of human EM tissue sections for the evaluation of MCs presence. Black arrows indicate MCs. **(F)** Toluidine blue staining of secretive uterine endometrium revealed the absence of MCs. **(G, H)** Immunohistochemical analysis of C3a in EM tissue sections. AEC (red) chromogen was used to visualize the binding of anti-human C3a antibodies. AEC (red) chromogen was used to visualize the binding of anti-human C3a antibodies. Nuclei were counterstained blue with Harris Hematoxylin; scale bars, 50μm. **(I)** Immunoistochemical analysis of C3a in secretive uterine endometrium revealed the absence of C3a.

C3a is one of the most important stimuli for MC activation (**Figure 3A**). Thus, we measured the levels of C3a in the PF of EM patients (n = 16), and compared them with those obtained from myoma and fibroma patients (women undergoing laparoscopy, without alterations of peritoneal cavity environment, n = 8). Our results showed that the PF of EM patients had significantly higher level of C3a compared to the control patients (**Figure 3B**). Furthermore, we investigated the target molecule of C3a, namely C3aR, through GEP analysis. The expression of this gene partially mirrors that of C3, with high levels in EM lesions and low expression in the eutopic endometrium (**Figure 3C**). We confirmed the C3aR expression in EM lesions by IHC (**Supplementary Figure 8**).

Hypothesizing that C3a present in the peritoneal cavity of EM patients could stimulate the MCs within the EM lesions, we investigated the pattern distribution of MCs in the EM tissue. We therefore stained human EM tissue sections with toluidine blue to highlight MCs presence in the tissue. The histochemical analysis confirmed that EM lesions were rich in MCs (**Figures 3D, E**), compared to normal endometrium (**Figure 3F**). The IHC for C3a on the same sections corroborated the presence of this anaphylatoxin in EM lesions as well (**Figures 3G, H**) and revealed the absence in normal tissue (**Figure 3I**).

### C3a is Involved in an Auto-Amplifying Loop of Inflammation Between MCs and EM Cells

In order to mimic the cross-talk between EM cells and MCs and understand the role of C3a, we set up a co-culture assay, seeding endometrial cell line (AN3CA) at the bottom of a 24-well plate and placing the HMC-1 (a MC cell line) onto the upper chamber of a transwell system (1 µm diameter pores), in the presence or absence of a pool of EM-PF and/or a blocking anti-C3a antibody. Stimulation with TNF-α was used as a positive control.

**Figure 4:**
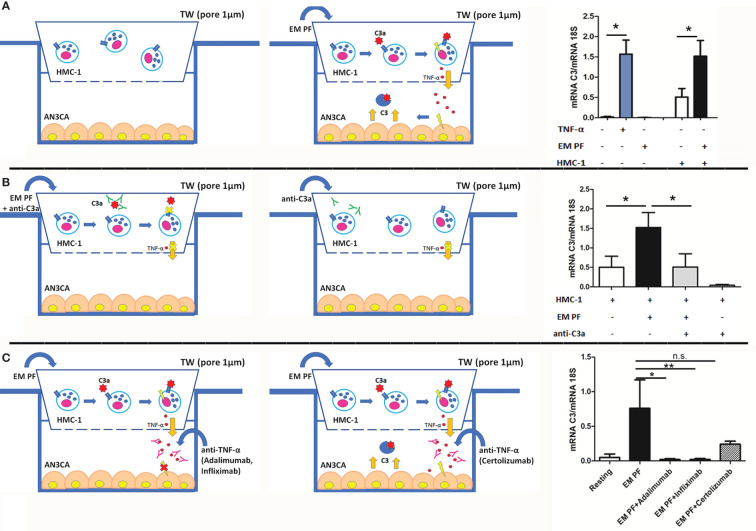
The co-culture of MCs with endometrial cells, in the presence of EM-PF, induced the expression of C3, which was inhibited by C3a blocking antibody. **(A)** The C3 gene expression was evaluated by RT-qPCR on endometrial AN3CA cells alone (resting conditions), stimulated with TNF-α (+TNF-α) or with a pool of EM peritoneal fluid (+EM-PF); or co-cultured with MCs alone (+HMC-1), or with EM-PF (+HMC-1+ EM-PF). Similar experiments were performed in the presence of anti-C3a blocking antibody **(B)**, or in the presence of anti-TNF-α blocking antibodies [(Infliximab, Adalimumab or Certolizumab), **(C)**]. In the left part of the figure the graphical representations of the blocking co-culture experiments. Data are expressed as mean of three independent experiments conducted in double ± standard error. *p < 0.05, **p < 0.01, n.s., not significant.

AN3CA cells, cultured with HMC-1 alone, expressed low levels of C3. However, after HMC-1 stimulation with EM-PF, AN3CA cells began to express higher quantities of C3 (**Figure 4A**). Surprisingly, stimulation with EM-PF alone (in absence of HMC-1) was not sufficient to increase C3 expression levels, in the absence of HMC-1. A significant level of C3 production by AN3CA was only evident following addition of HMC-1 stimulated by EM-PF, showing C3 levels comparable to TNF-α stimulation (positive control). Pre-incubation of EM-PF with a blocking monoclonal anti-C3a antibody completely abrogated the effect of EM-PF stimulation, bringing C3 levels to the resting values (**Figure 4B**).

With a view to gauge a potential novel immunotherapeutic approach for EM, we investigated in our co-culture model the effect of three different anti-TNF-α antibodies. Infliximab and Adalimumab (both in clinical use) showed a strong effect in blocking C3 expression by AN3CA, whereas Certolizumab did not block significantly the effects induced by the EM-PF on HMC-1 cells (**Figure 4C**).

## Discussion

Dysregulation of the complement system has largely emerged as an important mechanism in the pathogenesis of EM (32, 33), regulating chronic inflammation and concurring as a precipitating factor in EM-associated ovarian cancer (13). Several complement components such as C3, C4A, C7, factor D, factor B, factor H, and mannose-associated serine protease 1 (MASP1), are differentially expressed in EM compared to normal uterine tissues (13). The most studied protein is C3 (34), although its contribution in EM pathogenic mechanisms has not been elucidated yet.

The presence of C3 in EM tissue was first highlighted in 1980 (35). Subsequent studies confirmed the presence of higher levels of C3 in the EM lesions (13, 18, 19, 32, 33, 36–39). In the current study, C3 was found to be widely distributed in the EM lesions with variable intensity, being mostly localized in the glandular-like structures and in the vessels. Despite detecting the presence of C3 in both eutopic and ectopic endometrium (**Figure 1**), we noted a considerably stronger positivity of C3 in ectopic endometrium of the ovary and the abdominal wall.

Different C3 expression levels between ectopic tissues and normal uterus were also confirmed by RT-qPCR on mRNA samples extracted directly from ovarian EM cysts (**Figure 1F**) and primary cells isolated from the same district (**Figures 1G, H**). In fact, C3 appeared to be produced locally and in higher amounts in the ectopic tissues compared to the normal uterus. To gain a more complete insight into the EM microenvironment, we also considered the potential contribution of endothelial cells isolated from EM lesions in the production of C3. However, we found out that these cells were not responsible for the differential expression of C3 transcript observed between EM tissue and normal uterus.

Another innovative element in the present study was the attempt to elucidate the factors responsible for the increased C3 expression by the ectopic endometrial cells as compared to the uterine endometrium (36). In fact, we hypothesized that the inflammatory microenvironment found in EM lesions and in the abdominal cavity might influence the local C3 production. A potential candidate for the upregulation of C3 expression by endometrial cells is the PF rich in pro-inflammatory factors (39). In particular, TNF-α concentrations in the PF are elevated in EM patients and its concentration correlates with the severity and stage of the disease (40, 41). Furthermore, TNF-α and IL-1β are known to be elevated in EM *milieu* (39, 42). We confirmed that the stimulation of endometrial cells with TNF-α (100 ng/mL), and to a lesser extent with IL-1β (5 ng/mL), induced an enhanced production of C3.

Based on these findings we set up an *in vitro* model of EM to understand the pathogenic mechanisms through monitoring C3 expression by AN3CA cells under different conditions. Surprisingly, the stimulation of normal endometrial cells with EM-PF did not directly cause an increase in C3 gene expression, suggesting that the concentration of TNF-α in the PF (around 50 pg/ml) was not sufficient for a direct endometrial cell activation.

Speculating on the source of an increased concentration of TNF-α in the endometrial tissue, we examined a *C3*^-/-^ mouse model. Interestingly, no studies involving complement-deficient animal models have been reported so far. Our mouse model allowed us to primarily demonstrate that C3 deficient mice were refractory to the development of endometriotic lesions, whereas WT mice developed a higher number of EM cysts (**Figure 2B**), suggesting that the lack of C3 prevented the EM cyst formation. It is interesting to note that in a syngeneic mouse model, which involved injection of normal uterus (and not EM lesion) in the peritoneum, one would not expect availability of a reasonable amount of locally synthesized C3. However, we still noticed statistically significant differences in cyst formation, indicating that the circulating C3 probably plays an important role in this case. Moreover, we accidentally discovered the involvement of MCs in the pathogenic mechanisms concerning C3, since the WT mice were characterized by the presence of degranulated MCs in PF, as demonstrated by the detection of a considerably higher amount of tryptase, compared to *C3*^-/-^ mice (**Figure 2F**).

It is largely considered that macrophages, among various immune cells, exert a pivotal role in the pathogenesis of EM (22, 43, 44). These cells could be responsible for the local synthesis of C3 in EM lesions as well (45), but, since we did not notice differences in mouse peritoneal washings and in co-culture experiments (**Supplementary Figure 9**), we decided to focus only on MCs.

Several studies have demonstrated the involvement of MCs in the EM lesion formation and progression (46, 47). In particular, a recent study showed that the number of total MCs, as well as activated MCs, was significantly increased in EM lesions in both animal models and humans. An increased presence of activated and degranulated MCs in deeply infiltrating EM and its close histological relationship with nerves strongly suggest that MCs contribute to the development of pain and hyperalgesia in EM, possibly by a direct effect on the nerve structures (11). Based on this growing evidence, the use of MC stabilizers and inhibitors has also been proposed as a promising treatment for EM and its associated pain (11, 47). The diffuse infiltration of numerous MCs, accompanied also by increased presence of scattered granules, has been frequently observed throughout the stromal lesions, whereas in the eutopic endometrium and normal uterine serosa of the EM patients and controls, MCs were rarely detected (12). Thus, in our study, EM lesions were found to be rich in MCs (**Figures 3D, E**), compared to normal endometrium (**Figure 3F**), as determined by toluidine blue histochemical staining. A good proportion of MCs were localized around blood vessels and in the fibrotic interstitium of endometrial cysts, suggesting a particularly close relationship of MC localization with fibrosis and adhesion (48).

The point of connection between C3 and MCs in EM microenvironment could be the presence of C3a, one of the most important stimuli for MC activation (49). C3a formation is due to complement activation in the EM microenvironment, previously described by the presence of different complement components and complement activation products, such as C1q, MBL, C1INH, C4 and C3c SC5b-9, in the PFs and in the sera of EM women (14, 50). One of the most reasonable explanation could be the setting off of the coagulation cascade, which is caused by the typical periodic bleeding in the EM tissue. Interaction between complement, coagulation and contact systems are well-established. Thrombin, FXIa, FXa, FXa and plasmin cleave C3 (and C5) to C3a and C3b; activated platelets are also involved in C3 cleavage (51–53). Another activator of the C3 is heme that is released from hemoglobin during hemolysis; heme induces deposition of C3 fragments on the erythrocytes (54). Alternative pathway activation can occur through its up-regulator, properdin, binding to activated platelets promoting C3(H_2_O) recruitment and complement activation (55). In addition, stimulation of endothelial cells by C3a or other factors promptly induces expression of P-selectin, which by binding to C3b, induces the formation of C3 convertases (56). Recently, MASP-2 has been shown to cleave C3 in the absence of C2 and C4, in addition to and cleaving prothrombin into active thrombin (57–59).

We confirmed the presence of higher levels of C3a in the EM-PF compared to control non-EM healthy women (**Figure 3B**); we also detected C3a in EM tissue samples but not in normal endometrium (**Figures 3G–I**). Moreover, in the co-culture assay, the pre-incubation of EM-PF with a blocking monoclonal anti-C3a antibody completely abrogated the effect of EM-PF stimulation, bringing C3 levels to resting values.

C3a present in the EM-PF can act, through C3aR interaction, which is abundantly expressed on the MCs present in the EM tissue (10, 36). C3a involvement in EM has also been suggested by previous evidence highlighting its correlation with chemokine CCL8 (60), a promotor of the cross-talk between endometrial cells and MCs in the development of EM through the binding to its receptor CCR1, characterized by over-expression in the ectopic endometrium and colocalization with blood vessels in ovarian endometriomas (61).

## Conclusions

In conclusion, using *C3*^-/-^ murine model of EM, we demonstrated a pivotal role of C3 in the progression of EM lesions. It appears normal endometrial cells, under pro-inflammatory stimuli, start to produce C3. C3a, locally produced by complement activation and stimulated by the pro-inflammatory *milieu*, seems to recruit and activate MCs in the EM lesions, which can have a pathogenic consequence in the ectopic EM by releasing histamine and TNF-α as well as additional inflammatory factors. C3 appears, therefore, as a central factor in a regulatory feed forward loop, which is able to amplify the inflammatory microenvironment, in which the MCs are protagonists. This study opens up a new window for the identification of novel therapeutic targets for treating EM.

## Data Availability Statement

The original contributions presented in the study are included in the article/**Supplementary Material**. Further inquiries can be addressed to the corresponding author.

## Ethics Statement

The studies involving human participants were reviewed and approved by The Regional Ethical Committee of FVG (CEUR), Udine, Italy (Prot. 0010144/P/GEN/ARCS 2019). The patients/participants provided their written informed consent to participate in this study. The animal study was reviewed and approved by The Institutional Animal Care Committee of the University of Trieste (Prot. 35/2010B).

## Author Contributions

CA, SZ, GR, and RB designed the experiments. CA, AB, VB, and SZ performed the experiments. PM, GZ, AMar and FR contributed crucial reagents/analytic tools. BB, MT, GM, and AMan analyzed data. CA, GR, and RB conceived the study. RB and GR supervised the study. CA, SZ, BB, and AB supervised experiments. CA, AB, UK, AMan, and RB wrote and revised the manuscript. All authors contributed to the article and approved the submitted version.

## Funding

This research was supported by grants from the Ministry of Health: Project code: ENDO-2020-23670288 “Pathogenesis of endometriosis: the role of genes, inflammation and environment” and by the Institute for Maternal and Child Health, IRCCS Burlo Garofolo, Trieste, Italy (RC20/16, RC23/18 to GR and 5MILLE15D to CA) and PORFESR 2014/2020 FVG (“TiCheP” project) to RB.

## Conflict of Interest

The authors declare that the research was conducted in the absence of any commercial or financial relationships that could be construed as a potential conflict of interest.

## Publisher’s Note

All claims expressed in this article are solely those of the authors and do not necessarily represent those of their affiliated organizations, or those of the publisher, the editors and the reviewers. Any product that may be evaluated in this article, or claim that may be made by its manufacturer, is not guaranteed or endorsed by the publisher.
