# Supplementary material for: The Inflammatory Feed-Forward Loop Triggered by the Complement Component C3 as a Potential Target in Endometriosis
Source: Front Immunol. 2021 Aug 13;12:693118. doi: 10.3389/fimmu.2021.693118 (PMC8418148; doi:10.3389/fimmu.2021.693118)
Supplement: Supplementary file 1 [file DataSheet_1.pdf]

Supplementary Material

1.1 Supplementary Figures

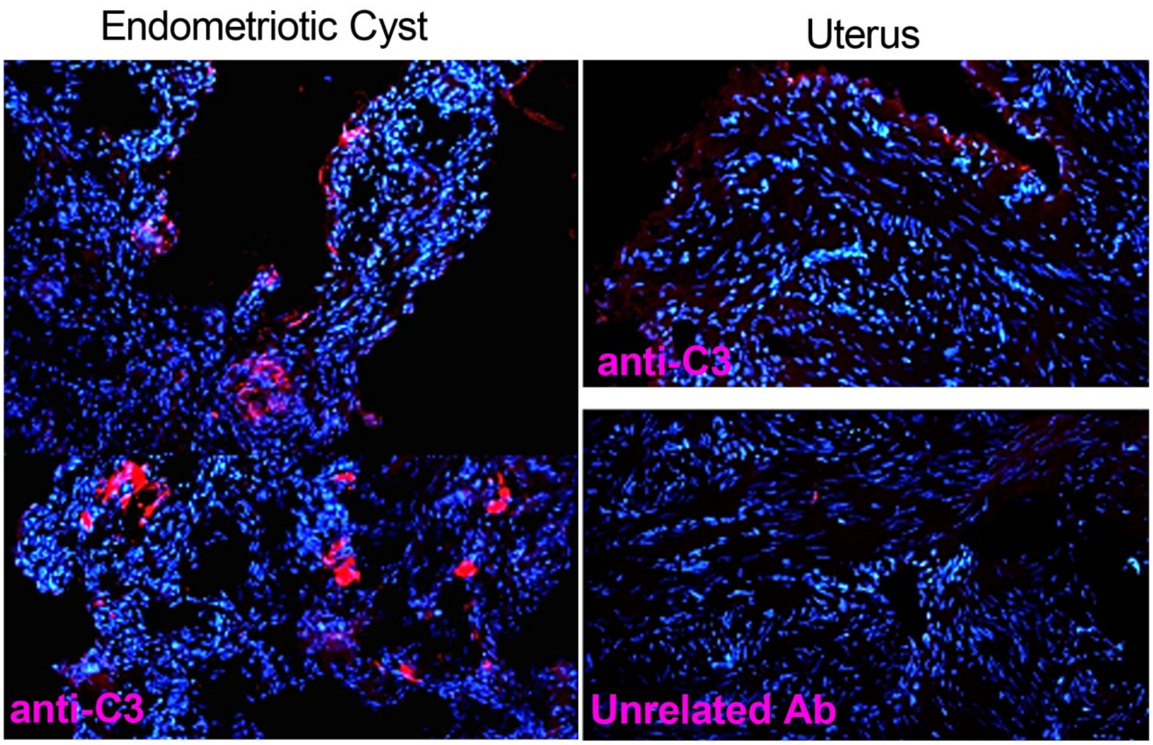

Supplementary Figure 1. Immunofluorescence localization of C3 in endometrial eutopic (Uterus, right part) and ectopic tissue (Endometriotic Cyst, left part) using frozen sections. C3 stained (in red; with goat anti-human C3; Quidel, Rome, Italy) strongly in endometriotic cyst section compared to normal uterus. The nuclei were counter-stained in blue with DAPI (original magnification 100 x).

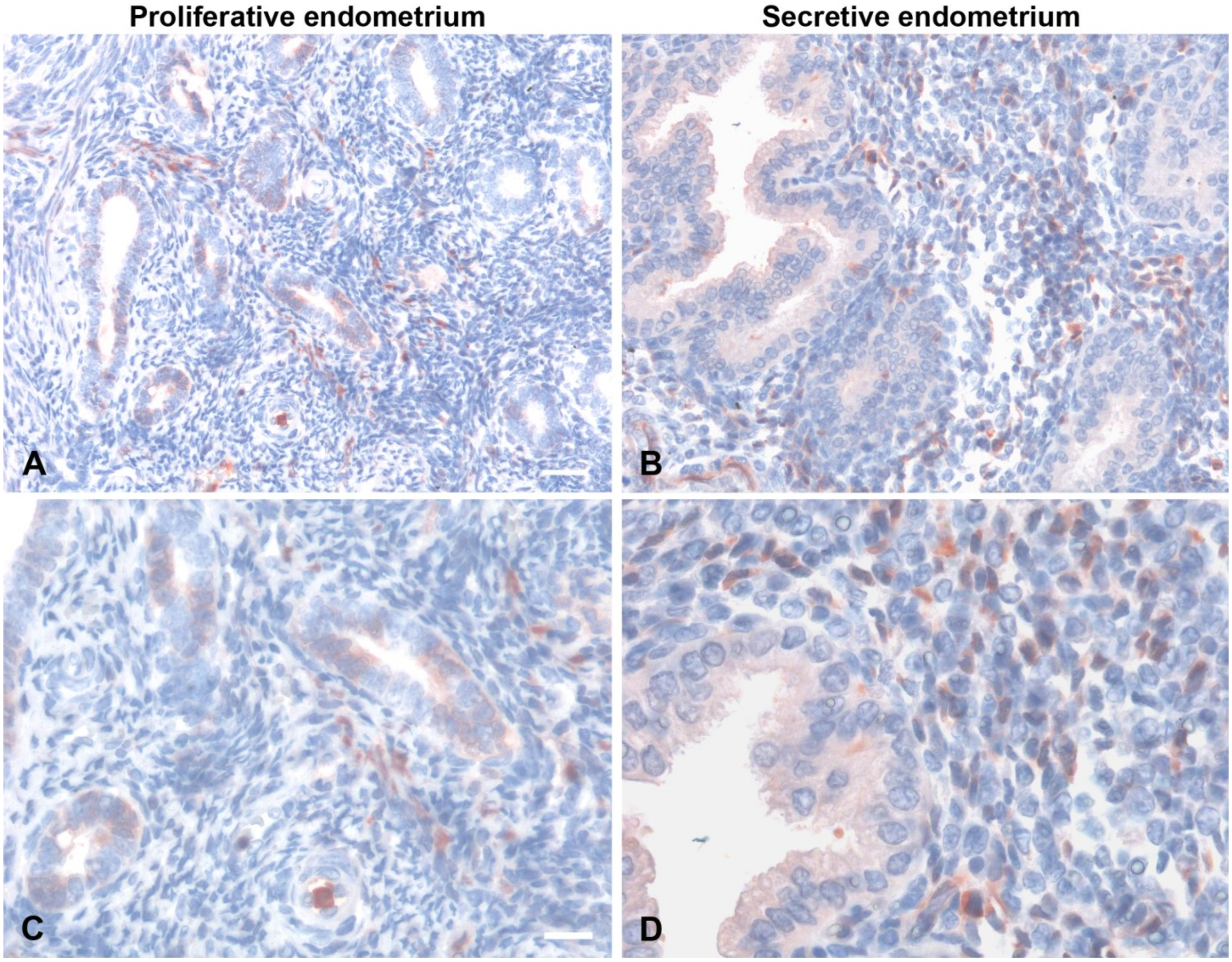

Supplementary Figure 2. Representative microphotographs showing C3 IHC staining in proliferative (A, C) and secretive (B, D) endometrium. AEC chromogen was used to visualize the binding of anti-human C3 antibody; (A, B) original magnification 200 x, scale bar 50 µm; (C, D) original magnification 400 x, scale bar 100 µm.

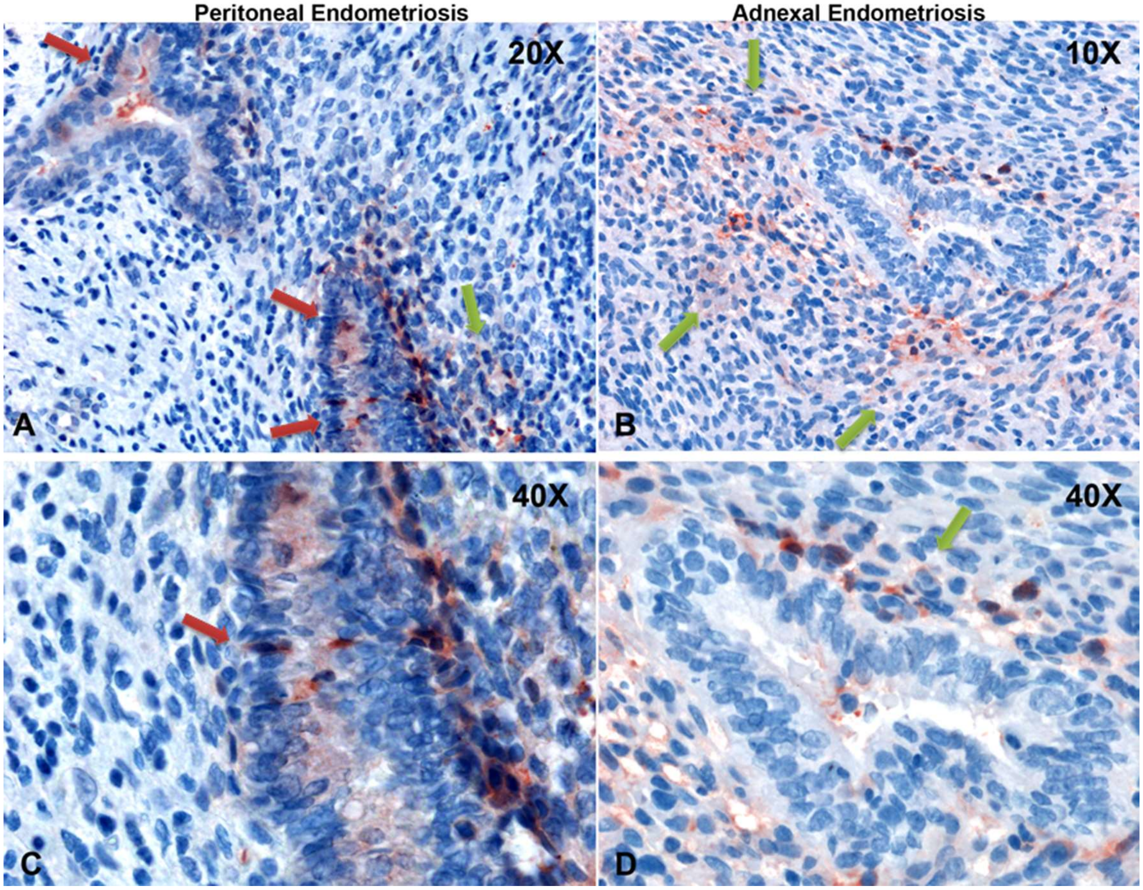

Supplementary Figure 3. C3 expression by human ectopic endometrial tissue. Representative microphotographs showing expression of C3 by IHC in abdominal wall (A, C) and adnexal (B, D) endometriosis (EM). Red and green arrows represent C3 positivity, localized in the glandular-like structures and in the cytogen stroma, respectively. AEC (red) chromogen was used to visualize the binding of anti-human C3 antibodies. Nuclei were counterstained in blue with Harris Hematoxylin.

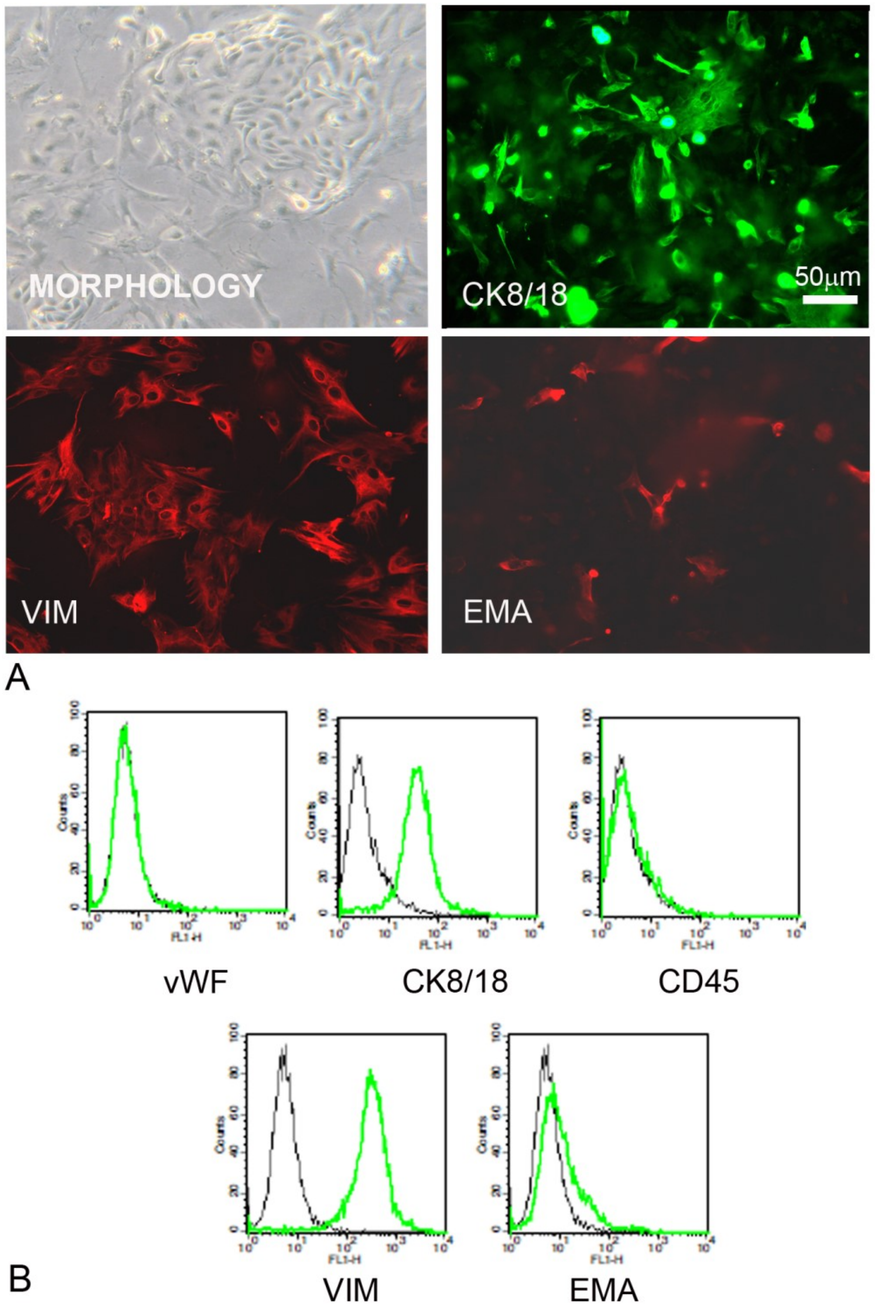

Supplementary Figure 4. (A) Morphological features of endometrial cells isolated from EM cyst biopsies. Images were acquired by phase-contrast microscope (Leica original magnification: 200 ×). Endometriotic cells were characterized by immunofluorescence for the expression of cytokeratin 8/18 (CK) in green, vimentin (VIM) and Epithelial Membrane Antigen (EMA) in red. The cells were grown to confluence in 8-chamber culture slides. After fixation and permeabilization, the cells were stained with primary mAb followed by anti-mouse-FITC or Cy3 conjugated F(ab)′ secondary antibodies. Original magnification 200 ×. (B) Expressions of von Willebrand Factor (vWF), CK8/18, CD45, VIM and EMA were analyzed by cytofluorimetric analysis. The expression of these markers (green lines) was compared with appropriate control antibodies (black lines).

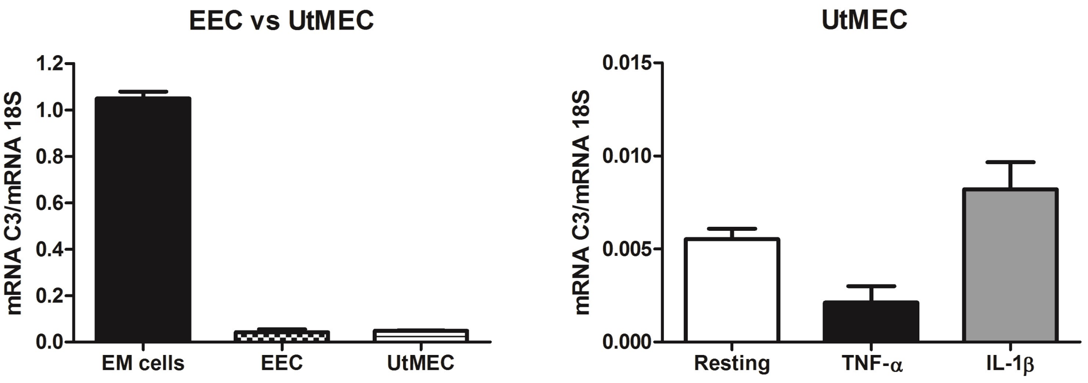

Supplementary Figure 5. (A) C3 mRNA expression was evaluated by RT-qPCR in Endometriotic Endothelial Cells (EEC, n = 4) and compared to Uterine Microvascular Endothelial Cells (UtMEC, n = 3). Endometrial cell isolated from EM cyst (EM cells) were used as a calibrator. (B) UtMECs were stimulated overnight with TNF-α (100ng/mL) or IL-1β (5ng/mL), and the C3 expression was analyzed by RT-qPCR. No significant modulation of C3 expression was observed. Data are expressed as mean ± standard error.

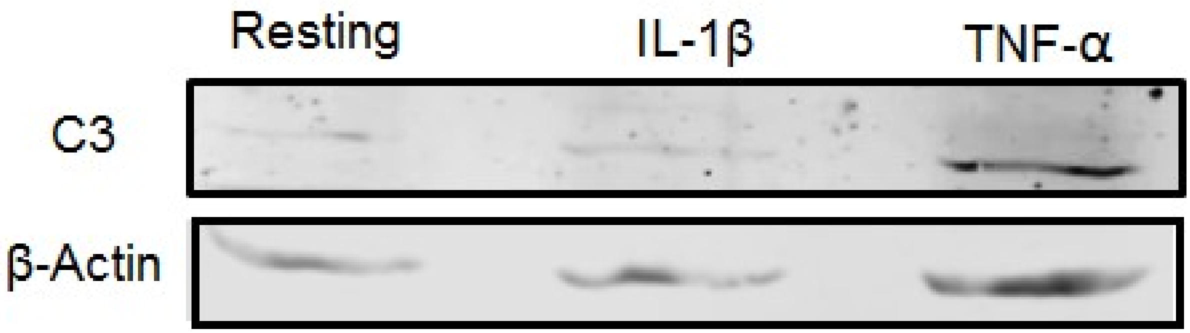

Supplementary Figure 6. Stimulation of AN3CA cells with pro-inflammatory cytokines up-regulated C3 expression. AN3CA cells were stimulated overnight with TNF-α (100 ng/mL) or IL-1β (5 ng/mL), and C3 expression was evaluated in cell lysates by western blot (WB) via Odyssey-LICOR scanner.

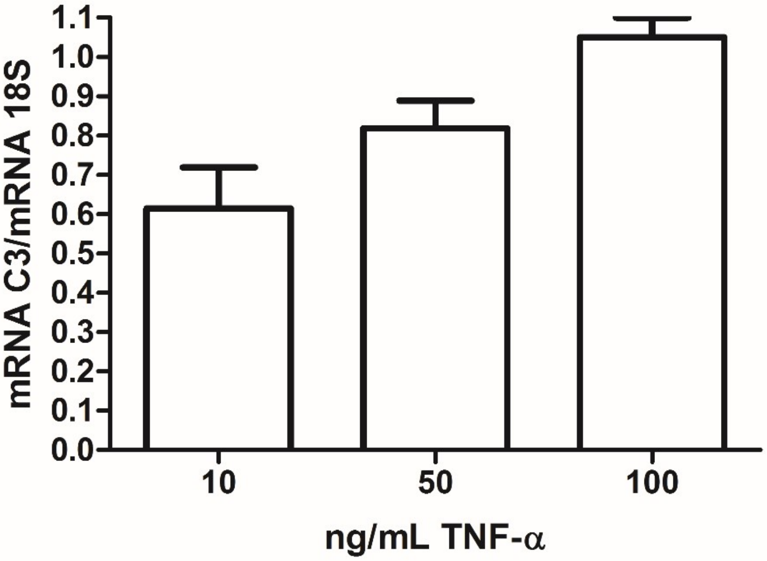

Supplementary Figure 7. TNF-α induced expression of C3 mRNA by AN3CA in a dose-dependent manner. C3 mRNA expression was measured by RT-qPCR in AN3CA cells after 24 h stimulation using increasing concentrations of TNF-α (10, 50, 100 ng/mL). Data are expressed as mean ± standard error.

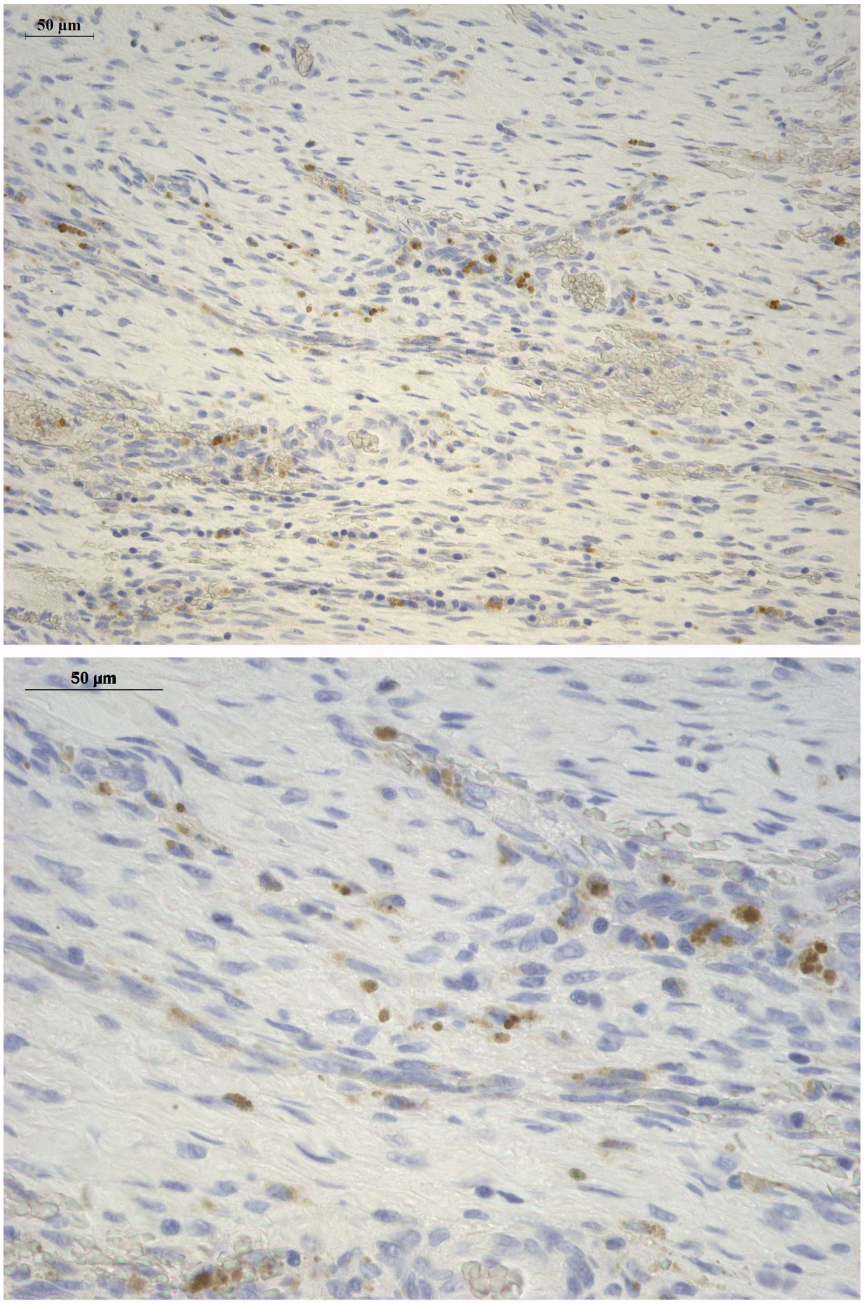

Supplementary Figure 8. C3aR expression by human ectopic endometrial tissue. Representative microphotographs showing expression of C3aR by IHC in ovarian EM lesion. DAB (brown) chromogen was used to visualize the binding of anti-human C3aR antibody. Nuclei were counter-stained in blue with Harris Hematoxylin. Scale bar 50 µm.

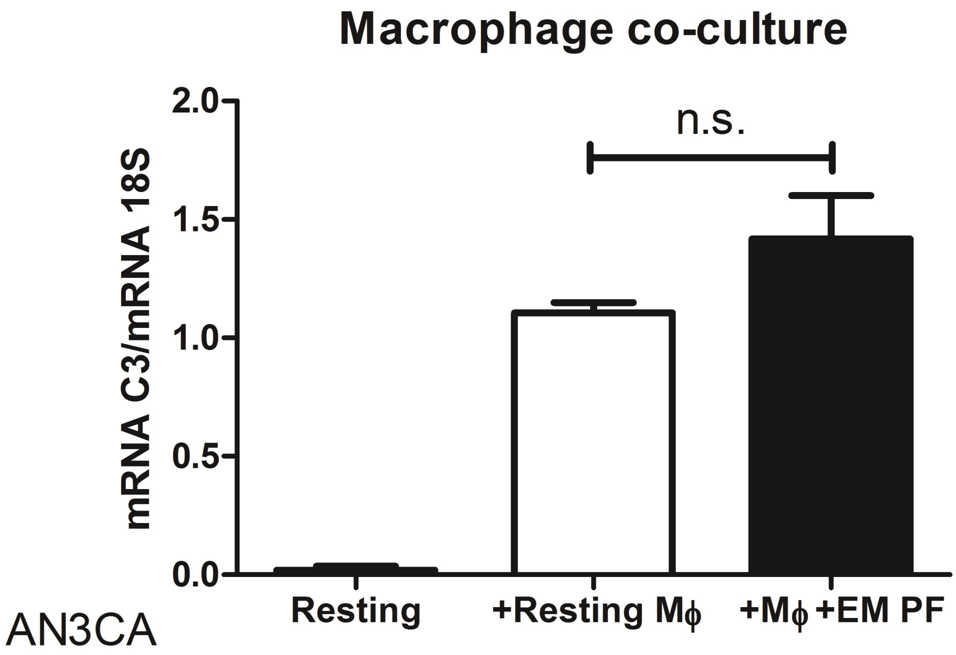

Supplementary Figure 9. Co-culture of THP-1 cells [differentiated into macrophage-like phenotype (MΦ) by adding 15 ng/mL of PMA] with endometrial cells induced the expression of C3; however, the presence of EM-PF did not increase C3 expression. C3 gene expression was evaluated by RT-qPCR in endometrial AN3CA cells alone (resting conditions) and AN3CA co-cultured with THP-1 alone (+Resting MΦ), or with a pool of EM-PF (MΦ + EM-PF). No statistical differences were observed between the last two conditions.

1.2 Supplementary Table

Gene	Tm	Sense	Sequence	Accession number
**18 S**	61	Forward	ATCCCTGAAAAGTTCCAGCA	NM_022551.2
		Reverse	CCCTCTTGGTGAGGTCAATG	
**C3**	61	Forward	CCTGCTACTAACCCACCTCC	NM_4557385a1
		Reverse	AACAGTGACTGGAACATCCCC	
**C5**	60	Forward	ATGGGCCTTTTGGGAATACTTTG	NM_4502507a1
		Reverse	ACATGGCCTGAGGAGTAACTAA	

Supplementary Table 1. Primers used for RT-qPCR analysis.
